# Annotations

**Published:** 1907-10-19

**Authors:** 


### Annotations.

### Morphinomania and Alcoholism.

It is without question a decided gain that drug habits have within recent years been subjected to systematic study, and have come to be regarded as conditions worthy of the attention not only of the social reformer, but also of the pathologist and physician. For this result gratitude is due to several agencies, and more particularly, within the ranks of the profession, to the Society for the Study of Inebriety. The quarterly journal of this Society almost invariably contains several papers well deserving of attention, and presents evidence of a resolution to place the whole subject upon a secure scientific basis. In the current issue is the report of a discussion on "Alcohol and the Children of the Nation," which is introduced by a paper by Dr. Mary Scharlieb, and which includes contributions from Sir Jno. Gorst, Sir Jno. Kirk, Prebendary Carlile, Mr. Pearce Gould, Dr. Jno. F. J. Sykes, and others. The story is not pleasant reading, but that it rests on a substantial basis of fact cannot be doubted. Probably many will agree that the most hopeful aspect of the situation is to be found in Mr. Pearce Gould's contention that "the school must be made the saviour of the State." Related to this question is the attempt to cure the individual victim of drug habits by the use of medicines. Some months ago a report was circulated of the wonderful efficacy of a Malayan plant, *Combretum Sundaicum*, in the treatment of the opium habit. The Society for the Suppression of the Opium Trade has had the drug tried in this country under medical supervision, and has recently published a few case records which present encouraging results. Particulars will be supplied by the Society to any medical practitioner applying at the offices, 181 Queen Victoria Street, E.C.

### Proprietary Remedies in Australia.

In December 1906 the Australian Government appointed Mr. Octavius C. Beale as a Royal Commissioner to conduct an inquiry into the manufacture, sale and use of the so-called patent or proprietary medicines, the composition of which is not disclosed, and for which medicinal or remedial properties are claimed. The first volume of the Commissioner's report has now been issued, and whatever may be said of its details, one, at least, of its general conclusions will, it may be hoped, fasten itself upon the popular mind. This conclusion is found in the proposition that the preservation of secrecy and of the privilege to deceive is absolutely indispensable to the traders whose traffic is reported upon, and that the perpetuation of the advantages they now enjoy means moral corruption, physical deterioration, and national decadence. Turning to details we are told, and evidence is advanced for the statements, that abortifacient poisons and potent drugs are announced and sold widely, though under a thin disguise; that the natural phenomena of healthy puberty are utilised with satanic ingenuity to frighten young men and women into the clutches of quacks; that opiates for infants and children are sold without practical restriction; and that for the adolescent and adult there is a complete set of "cure systems" in which may be traced abundant evidence of "trickery, treachery, humbug and fraud." The whole of the practices in question are vigorously denounced as constituting "a carcinomatous inversion of the natural activities and of the principles of industry," and they are presented as "incapable of conversion" and needing to be "removed." The recommendations are not less vigorous. They include the compulsory publication of the qualitative and quantitative composition of all proprietary remedies; the prohibition of all advertisement of such remedies; and the establishment for purposes of control of a government bureau of chemistry. At the same time it is suggested that letters patent should continue to be granted for any approved and novel formulæ for the prevention, alleviation or cure of human ailments, provided that such formulæ shall, in the opinion of the Health Office, justify the claims of the inventor. Whether Australia will adopt this advice remains, however, to be seen.

### The Quarterly Journal of Medicine.

The first issue of this new journal has just appeared, and it may be cordially welcomed as an attempt to encourage and advance the study of clinical medicine. It appears to be promoted by the recently established Association of Physicians of Great Britain and Ireland, a brief account of the first annual meeting of which appears in its pages. The main purpose of the new journal would seem to be the provision of an opportunity for the publication of clinical investigations of a very full and elaborate character. The extent of an author's demands in this direction is sometimes so great that the ordinary journals cannot make provision to meet it. Further, it must be admitted that many of these investigations necessitate attention to so many details that their record readily fails to appeal to those engaged in the daily anxieties of practice. Yet sooner or later upon work of this order are grafted methods of diagnosis and treatment of the highest possible value. Hence, there may be found an ample justification for the appearance of our latest contemporary. When, further, it is stated that the chief director of the editorial department is Professor Wm. Osler, enough has been said to command the confidence and ensure the goodwill of the profession. Of recent years the work of the surgeon has assumed such an extension of its domain that the medical aspects of practice have been to some extent obscured. The new Association and the new journal will do something to rectify this position, and they are therefore worthy of all encouragement. In the first issue of the journal the papers are all of a high order and of great interest, and the illustrations are of quite exceptional value. To mention them all is impossible, and amidst so much that is worthy of praise selection seems somewhat invidious. Perhaps, however, we may be permitted to refer to the article on "Multiple Hereditary Telangiectases with Recurrent Epistaxis," by Professor Osler, and to that on "Suprarenal Sarcoma in Children," by Dr. Robert Hutchison.

